# Fecal Immunochemical Testing for Colorectal Cancer Prevention in Two Public Hospitals

**DOI:** 10.1007/s12029-025-01187-y

**Published:** 2025-02-22

**Authors:** Changlin Gong, Maria Teresa Medina Rojas, Maria Gabriela Rubianes Guerrero, Michail Kladas, Arameh Mousakhanian, Aarushi Sudan, Adejoke Johnson, Kimberly Cartmill, Elana Sydney, Donald P. Kotler

**Affiliations:** 1 Department of Internal Medicine, Jacobi Medical Center, Albert Einstein College of Medicine, Bronx, NY USA; 2 Department of Internal Medicine, Metropolitan Hospital/New York Medical College, New York, NY USA; 3 Division of Gastroenterology and Hepatobiliary Diseases, Metropolitan Hospital/New York Medical College, New York, NY USA; 4 Gastroenterology, Hepatology, and Nutrition Service, Memorial Sloan Kettering Cancer Center, New York, NY USA; 5 Department of Internal Medicine, North Central Bronx Hospital, New York, NY USA; 6 Division of Gastroenterology, Jacobi Medical Center/Albert Einstein College of Medicine, 1400 Pelham Parkway South, Bronx, NY 10461 USA

**Keywords:** Fecal immunochemical testing, Colorectal cancer, Cancer prevention, Cancer education, Colonoscopy

## Abstract

**Introduction:**

The fecal immunochemical test (FIT) is highly effective in reducing colorectal cancer (CRC) mortality, but patient adherence to the screening process remains questionable. We present preliminary findings from a quality improvement (QI) initiative, in order to assess screening adherence and findings.

**Methods:**

All FIT specimens in a 30-month period were retrospectively examined. Patients with positive results were included, and information was collected via electronic medical record, including QI measures such as colonoscopy completion and findings. All data were de-identified. Patients were divided into “asymptomatic group” and “symptomatic group” based on clinical manifestations. Adherence and findings were analyzed.

**Results:**

FIT results were positive in 174 out of 2400 specimens. Colonoscopy was performed in 47.6% of all FIT-positive cases after a median interval of 5.5 (interquartile range, IQR 3–10) months, with 10% having CRC, 51.3% having adenomas, and 17.5% having advanced adenomas. Of all nine patients who had CRC, seven were in the symptomatic group. All five advanced cancers were found in the symptomatic group. Patients who actually completed colonoscopy were significantly younger than those who did not (median 61.5 years, IQR 56.5–69 years, vs. 64.5 years, IQR 59–71 years, *P* = 0.048). Patient-related reasons, primarily refusal, accounted for 65.9% of unperformed colonoscopies. No significant difference was found in adherence and yield between asymptomatic and symptomatic groups.

**Conclusion:**

Prevalence of colorectal adenomas and cancers is high in FIT-positive patients. A substantial number of CRCs and potentially preventable CRCs must have been missed because of low adherence rate, especially in older patients. Improving adherence to CRC screening in public hospitals requires enhanced patient engagement.

## Introduction

Colorectal cancer (CRC) remains the second leading cause of cancer deaths worldwide [1] although randomized controlled trials (RCT) and observational studies on fecal immunochemical tests (FIT) and colonoscopies have shown significant reductions in CRC mortality [2]. According to the Centers for Disease Control and Prevention (CDC), only 62.5% of the eligible population underwent a CRC screening process consistent with the United States Preventive Services Task Force (USPSTF) [3]. An estimate of 46–63% of deaths from CRC can be attributed to failure to receive screening tests [4]. Since colonoscopy can also remove adenomas, which carry a risk of progression to cancer, adherence to the CRC screening process can also reduce CRC incidence by 26–40% [5]. Therefore, promoting adherence to the CRC screening process on a population level is essential in decreasing CRC incidence and mortality.

Socioeconomic disparity was also noted in CRC epidemiology, disease burden, and mortality, and one of the causes for worse outcomes in people with lower socioeconomic status was lower participation in CRC screening [6]. Public hospitals of the United States (US) mainly serve people with relatively low socioeconomic status. They provide continuity care for working-class patients as well as emergent and/or inpatient care for any patient in need, regardless of their ability to pay. Failure to adhere to CRC screening in public hospitals has been observed in clinical practice, which can increase the risk of cancer development, delay care, and worsen disparity in this already underprivileged population. Thus, we carried out this study to examine data on patient adherence, outcomes, and their clinical implications regarding CRC screening among an underserved population.

## Methods

This is an analysis of a preliminary quality improvement (QI) initiative in two public hospitals, which aimed at preventing CRC by promoting adherence to CRC screening, including adherence to colonoscopy after a positive FIT test. In the hospitals where we practice, no standardized patient education program was in place for CRC screening. Primary care providers perform patient education at their own discretion. Then, FIT kits are mailed or directly given to eligible patients in the primary care clinic.

A total of 2400 adult patients who completed FIT in primary care clinics between July 31, 2019, and December 31, 2021, were retrospectively reviewed. Patients with positive FIT results were identified. As we examined the electronic medical record (EMR) of these patients, we noticed that some FIT kits had been distributed to patients with signs or symptoms of possible gastrointestinal disease. We divided the patients into “asymptomatic” and “symptomatic” groups, based upon the presence or absence of anemia (hemoglobin < 13.5 g/dL in male patients and < 12 g/dL in female patients), weight loss (unintentional decrease of 5% body weight or more within 6 months), or recent onset gastrointestinal symptoms suggestive of CRC, including diarrhea, constipation, rectal bleeding, and abdominal pain.

Clinical information was obtained from EMR (EPIC, Verona, Wisconsin) and then de-identified, including demographic data and variables that will be used as measures in the QI initiative. Process measures included colonoscopy order and completion, reasons for not undergoing colonoscopy, as well as interval between the positive FIT result and colonoscopy. We classified the reasons for not undergoing colonoscopy into three categories: patient-related, system-related, and unclear. Patient-related reasons are defined as the absence of patient cooperation in order to complete colonoscopy, including patient refusal, patient relocating, patient seeing other providers, and patient not answering phone calls or not showing up to appointments. All other reasons are labeled as system-based, including colonoscopy scheduled but not yet completed, appointment cancelled because of coronavirus disease 2019 (COVID-19) pandemic, and failure to discuss colonoscopy. In this study, nonadherence is defined as failure to complete colonoscopy, regardless of the reason, while patient refusal means that colonoscopy was offered but the patient declined. Outcome measures included detection of polyps, adenomas, advanced adenomas, and cancers. For advanced adenoma, we used the latest definition from the National Comprehensive Cancer Network (NCCN): high-grade dysplasia, polyps ≥ 1 cm in size, or villous or tubulovillous histology [7]. Sessile serrated lesions were also classified into normal mucosa, hyperplastic polyps, or adenomas, based on the pathology report. Advanced cancer was defined as stage III or IV based on the tumor, node, metastasis (TNM) staging system of the American Joint Committee on Cancer (AJCC)/Union for International Cancer Control (UICC) [8]. Balancing measures included scope withdrawal time, cecal intubation rate, interval cancers (defined as a cancer diagnosis within 3 years of a colonoscopy that does not find cancer), and procedural complications. To further analyze the implication of positive FIT results, we compared the data between asymptomatic and symptomatic groups.

Statistical analysis was performed on R (version 3.6.3). Continuous variables were reported as median (interquartile range, IQR), and comparisons between groups were performed with the Mann–Whitney *U* test. Categorical variables were reported as number (percentage), and comparisons were made with the chi-squared test. Statistical significance is defined as *P* value less than 0.05. This is a QI project, and approval by the institutional review board was not required.

## Results

**Fig. 1:**
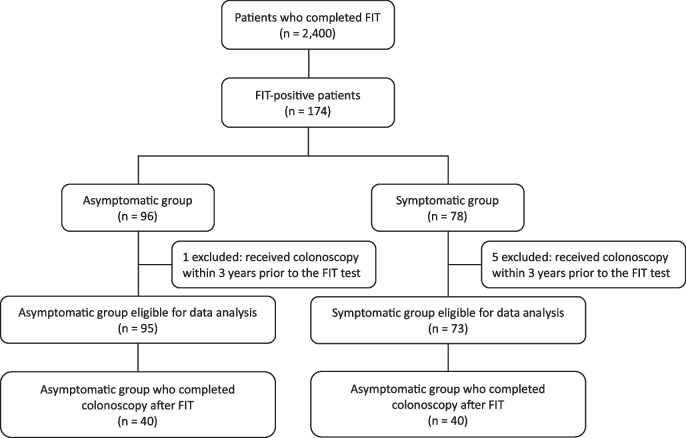
Flow diagram for patient inclusion. FIT, fecal immunochemical test

Among all 2400 FIT specimens, 174 (7.3%) were positive (Fig. 1). Among patients with positive FIT results, median age was 63 (57–70) years and 92 (52.9%) were female. Fifty-three (30.5%) self-identified as African-American, 32 (18.4%) as White, 13 (7.5%) as Asian, and 76 (43.7%) as others including Hispanic. Of all FIT-positive patients, 96 (55.2%) were included in the asymptomatic group, and 78 (44.8%) were included in the symptomatic group. There were no significant differences in age, sex, or race between the two groups (Table 1).

**Table 1:** Demographic characteristics of patients with positive FIT results

	Overall	Asymptomatic group	Symptomatic group	*P* value
No	174	96	78	
Age (years)	63 (57–70)	63.5 (58–69)	62.5 (56–71)	0.983
Sex				
Male	82 (47.1%)	44 (45.8%)	38 (48.7%)	0.705
Female	92 (52.9%)	52 (54.2%)	40 (51.3%)	
Race				
African-American	53 (30.5%)	25 (26%)	28 (35.9%)	0.414
White	32 (18.4%)	21 (21.9%)	11 (14.1%)	
Asian	13 (7.5%)	7 (7.3%)	6 (7.7%)	
Other (including Hispanic)	76 (43.7%)	43 (44.8%)	33 (42.3%)	

Continuous variables were reported as median (interquartile range (IQR)), and categorical variables were reported as number (percentage)

**Fig. 2:**
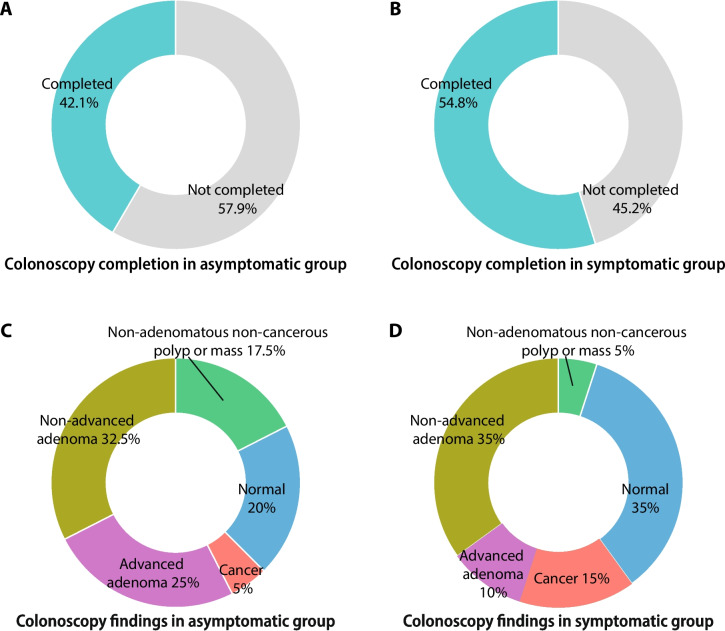
Summary of key results

One patient in the asymptomatic group and five in the symptomatic group received colonoscopy within 3 years prior to the FIT test and were thus excluded (Fig. 1). Follow-up colonoscopies were ordered in 132 (78.6%) patients overall, including 76 (80%) in the asymptomatic group and 56 (76.7%) in the symptomatic group. However, only 80 (47.6%) patients actually completed colonoscopy, including 40 (42.1%) in the asymptomatic group and 40 (54.8%) in the symptomatic group. No significant difference was found between groups in terms of colonoscopy order and completion (*P* = 0.607 and 0.103, respectively; Table 2 and Fig. 2). For those who failed to complete colonoscopy, patient-related reasons accounted for 65.9% of the cases, mainly patient refusal, while system-related issues were responsible in 23.9% of the cases. Median interval between positive FIT result and colonoscopy was 5.5 (3–10) months, and 57 patients (71.3%) who underwent colonoscopy did so within 9 months.

**Table 2:** Adherence and outcomes of colonoscopy in patients with positive FIT results

	Overall	Asymptomatic group	Symptomatic group	*P* value
No. eligible for analysis	168 (96.6%)	95 (99%)	73 (93.6%)	0.13
Colonoscopy ordered	132 (78.6%)	76 (80%)	56 (76.7%)	0.607
Colonoscopy completed	80 (47.6%)	40 (42.1%)	40 (54.8%)	0.103
Reason for nonadherence				
Patient-related	58 (65.9%)	35 (63.6%)	23 (69.7%)	0.562
System-related	21 (23.9%)	15 (27.3%)	6 (18.2%)	
Unclear	9 (10.2%)	5 (9.1%)	4 (12.1%)	
Interval between FIT and colonoscopy (months)	5.5 (3–10)	6.5 (3–9.5)	5 (2–10)	0.393
Patients receiving colonoscopy within 9 months	57 (71.3%)	30 (75%)	27 (67.5%)	0.459
Outcomes				
Any polyp or mass	58 (72.5%)	32 (80%)	26 (65%)	0.133
Adenoma	41 (51.3%)	23 (57.5%)	18 (45%)	0.263
Advanced adenoma	14 (17.5%)	10 (25%)	4 (10%)	0.077
Cancer	8 (10%)	2 (5%)	6 (15%)	0.116
Advanced cancer	4 (5%)	0	4 (10%)	0.116

Continuous variables were reported as median (interquartile range (IQR)), and categorical variables were reported as number (percentage)

A high proportion of patients had polyp or mass at colonoscopy, reaching 58 out of 80 (72.5%) overall. In the asymptomatic group, adenomas were detected in 23 cases (57.5%), 10 (25%) of which had advanced adenomas. In comparison, in the symptomatic group, 18 (45%) had adenomas, 4 (10%) of which were advanced. No significant difference in the prevalence of polyps or mass, adenomas, or advanced adenomas was found between asymptomatic and symptomatic groups (*P* = 0.133, 0.236, and 0.077, respectively; Table 2 and Fig. 2). Nine cancers were found among all 174 patients. One of the patients with advanced cancer (stage IIIB) was not included in the data analysis because advanced adenocarcinoma of the cecum and ileocecal valve was found before FIT (Table 2). The reason for FIT was not documented at that time. Eight of the 168 patients who were included in the data analysis were found to have cancer. Since 80 patients actually completed colonoscopy, the overall cancer detection rate was 10%. Six cancers were in the symptomatic group (cancer detection rate 15%), as compared to two cancers in the asymptomatic group (cancer detection rate 5%; *P* = 0.11; Table 2). Both cancers in the asymptomatic group were intra-mucosal adenocarcinomas (stage 0), while in the symptomatic group, one patient had intra-mucosal adenocarcinoma (stage 0), one had stage IIA adenocarcinoma, and four had advanced adenocarcinoma, including one stage IIIA adenocarcinoma, one stage IIIB adenocarcinomas, one stage IVA adenocarcinoma, and one stage IVB adenocarcinoma. Taken together, among all 174 patients, 5 advanced cancers were found, and all had signs or symptoms.

Balancing measures in this QI initiative mainly included quality indicators for colonoscopy. A total of 16 patients did not require biopsy, and their average withdrawal time was 13.2 min, consistent with the recommended time of ≥ 8 min [9]. Cecum was reached in 79 patients (98.8%), meeting the recommended target cecal intubation rate of 95% [9]. Adenoma detection rate (ADR) was 56.9% overall, 60% in males and 54.8% in females (different from Table 2 because the eight patients who had cancer were excluded from the denominators here), much higher than the recommended targets. For general population, the American College of Gastroenterology recommended a target ADR of 35% [9], while for FIT-positive patients, the USPSTF recommended target ADRs of 45% for men and 35% for women [10]. No interval cancer, defined as a cancer diagnosis within 3 years of a negative colonoscopy, was reported. No procedural complications occurred.

To identify people who are less likely to be adherent to colonoscopy, we compared demographic characteristic between patients who completed colonoscopy and those who did not. The median age for those who completed colonoscopy was 61.5 (56.5–69) years, significantly younger than those who did not (median 64.5 years, IQR 59–71, *P* = 0.048; Table 3), although caution is needed to interpret this result because the statistical significance is borderline and the sample size is relatively small. Among patients who completed colonoscopy, 40% were male, in comparison to 52.3% among those who did not. No significant difference in sex was found between the two groups (*P* = 0.11; Table 3). No significant difference was found in race either (*P* = 0.77; Table 3). We conducted logistic regression including four variables (age, sex, race, and symptoms) and confirmed this finding. Only age showed a significant correlation (*P* = 0.022 in univariable analysis and *P* = 0.018 in multivariable analysis including all four factors).

**Table 3:** Characteristics of patients who completed colonoscopy versus patients who did not

	Patients who completed colonoscopy	Patients who did not complete colonoscopy	*P* value
No	80	88	
Age (years)	61.5 (56.5–69)	64.5 (59–71)	0.048*
Sex			
Male	32 (40%)	46 (52.3%)	0.111
Female	48 (60%)	42 (47.7%)	
Race			
African-American	26 (32.5%)	25 (28.4%)	0.774
White	13 (16.3%)	19 (216%)	
Asian	5 (6.3%)	7 (8%)	
Other (including Hispanic)	36 (45%)	37 (42%)	

Continuous variables were reported as median (interquartile range (IQR)), and categorical variables were reported as number (percentage)

*Statistically significant

## Discussion

Our study confirmed high prevalence rates of adenomas, advanced adenomas, and CRCs in FIT-positive parents at the two public hospitals, regardless of symptoms, with prevalence rates similar in asymptomatic and symptomatic patients. More importantly, our study revealed a low adherence rate to colonoscopy in FIT-positive patients. In those who did complete colonoscopy, 51.3% had adenomas, 17.5% had advanced adenomas, and 10% had cancer, but over half of the FIT-positive patients did not complete colonoscopy, indicating that a substantial number of CRCs and potentially preventable CRCs may have been missed. The consequences can be disastrous. These results highlight the importance of adherence to a screening process in CRC prevention and early diagnosis.

Evidence supporting FIT as a screening tool for CRC is mainly from observational studies, but a study showed that CRC incidence decreased by 10% and CRC mortality decreased by 62% after the application of FIT [11]. In our study, over half of the FIT-positive patients had adenomas, 17.5% had advanced adenomas, and 10% had cancer. Previous reports of detection rates of adenomas, advanced adenomas, and cancers vary greatly among endoscopists, with the overall reporting detection rates of 39–73%, 12–35%, and 4–8%, respectively [12–20]. All these data confirmed that colonoscopy yield is very high in FIT-positive patients, and the cancer detection rate is even higher in our study, supporting its role in CRC screening. Another advantage of FIT is its timeliness. In our experience, the waiting time for a screening colonoscopy is frequently over 1 year. In contrast, FIT is the best immediately available option for CRC screening. Over 70% of the FIT-positive patients who completed colonoscopy were able to do it within 9 months. Delays of 10 months or more after a positive FIT were associated with a 48% increase in advanced-stage cancer [21, 22]. In public hospitals with limited resources, where the waiting list for colonoscopy is relatively long, prioritizing FIT-positive patients for colonoscopy can indeed facilitate the diagnosis of advanced adenomas or CRCs. Thus, FIT is a very valuable tool in optimizing resource allocation.

While current guidelines recommend FIT as a screening test for CRC [23], our study also examined the colonoscopy findings in patients with gastrointestinal symptoms and/or anemia. Seven out of nine CRC cases were found in the symptomatic group, although the higher prevalence compared to the asymptomatic group did not reach statistical significance, probably due to the relatively small sample size. In practice, symptomatic patients have a higher risk of CRC (0.5–40.6%, depending on the specific symptom [24]) and are usually recommended for colonoscopy directly. However, recent studies have shown that FIT has a good negative predictive value also in symptomatic patients [25, 26]. We did not include patients with negative FIT results, but future studies are needed to examine the positive and negative predictive values of FIT to determine the potential benefit of integrating FIT in the routine diagnostic workup for colorectal adenoma or cancer in symptomatic patients, including whether it can improve the timeliness of diagnosis and overall survival.

Patient adherence has always been an obstacle in all CRC screening modalities and one of the advantages of FIT compared to other modalities is improved patient adherence. A randomized controlled trial (RCT) found a higher adherence rate to fecal occult blood test (FOBT) compared to colonoscopy (67% vs. 38%) [27]. In addition to the superior sensitivity and specificity of FIT over traditional guaiac-based FOBT (gFOBT) [28], FIT also had better adherence (42.6–69% vs. 33.4–64%) [29–31]. One important reason is that gFOBT requires dietary restrictions because non-human hemoglobin may produce false-positive results and intake of vitamin C may produce false-negative results by inhibiting peroxidase activity [28]. Another possible factor that contributes to this difference is that gFOBT typically requires three samples on separate days and FIT requires one to two samples depending on the kit [31, 32]. Previous studies in the Midwest of the US, California, and China also reported a similar colonoscopy adherence rate of 40–50% after a positive FIT [33–35].

Previous studies explored different approaches to enhancing patient adherence. One promising strategy is shared decision-making. A multicenter RCT performed by the Polish Colonoscopy Screening Program, PICCOLINO study, saw an increase in participation in CRC screening by offering three different combinations of FIT and colonoscopy: screening colonoscopy only, screening colonoscopy at first and FIT for those who decline, as well as options for colonoscopy or FIT [36]. The latter two combinations resulted in 60 to 70% higher responses than colonoscopy only [36].

Our analysis on the reasons for nonadherence suggested that patient-related reasons accounted for 65.9% of the cases. Since there has not been a standardized educational program other than verbal communication by primary care providers in the two hospitals, we speculate that incomplete understanding of CRC screening and implications of positive FIT results might be the cause. One common misunderstanding among patients is that if they opt for FIT, then they do not need colonoscopy, and many fail to understand that a positive FIT mandates a follow-up colonoscopy to precisely localize and biopsy any suspicious lesions. However, this information is usually not documented on EMR. Thus, surveys are needed to assess patient understanding. Targeted interventions to both providers and patients might be helpful, such as enhanced pre-FIT education.

Other studies have shown success in increasing CRC screening rates through patient education and outreach. In Northern California, Kaiser Permanente increased the proportion of individuals up-to-date with screening from 38.9 to 82.7% and the proportion of colonoscopy completion from 41.1 to 83.1% between 2000 and 2015, through the use of direct-to-patient annual FIT outreach and education, with colonoscopy as a secondary option [33]. As patient participation increased, they found a 25.5% reduction in the annual incidence of CRC, a 52.4% reduction in CRC mortality, a 36% reduction in advanced CRC, and a 15% reduction in early-stage CRC [33]. In a community-based clinical linkage intervention to improve colorectal cancer screening among underserved Korean Americans, provision of FIT in addition to appropriate education significantly increased overall CRC screening and was better accepted than colonoscopy [37]. Another study in Texas also found that mailed outreach invitations significantly increased patient adherence [38]. Acceptance of different screening modalities may vary between ethnic groups: FIT acceptance is found to be higher among non-white populations while colonoscopy is better accepted by white participants [27]. Patient education can help bridge the gap between different ethnic groups. The Kaiser Permanente study reported that disparity in colon cancer incidence and mortality between non-Hispanic whites and blacks at the beginning of the study disappeared after implementing patient education [33].

## Limitations

This study has several limitations. First, some steps in the design may introduce selection bias. This is an electronic medical record-based retrospective study and only patients who returned FIT samples can be examined. The exact prevalence of adenomas and cancers in patients who did not complete colonoscopy cannot be accurately calculated so we can only estimate based on the data from those who completed colonoscopy. Second, the study was carried out in the midst of the COVID-19 pandemic, which may lead to higher risks of cancelation and loss to follow-up, increasing both patient-related and system-related nonadherence. Thus, the post-pandemic adherence rate might have been improved. Since some pre-pandemic studies reported similar adherence rate, the effect of pandemic is very likely limited [33]. Third, provider-specific data were not available, so differences among primary care providers were not analyzed. Finally, the sample size is relatively small, which may introduce random variation.

## Conclusion

Prevalence of advanced adenoma and cancer is high in a FIT-positive population. A substantial number of CRCs and potentially preventable CRCs may have been missed because less than half of FIT-positive patients completed colonoscopy, especially older patients. Standardized educational program on CRC screening might be essential to ensure that patients can benefit from CRC screening at a population level.

## Data Availability

No datasets were generated or analysed during the current study.
