# Correction: Fecal Immunochemical Testing for Colorectal Cancer Prevention in Two Public Hospitals

**DOI:** 10.1007/s12029-025-01223-x

**Published:** 2025-05-10

**Authors:** Changlin Gong, Maria Teresa Medina Rojas, Maria Gabriela Rubianes Guerrero, Michail Kladas, Arameh Mousakhanian, Aarushi Sudan, Adejoke Johnson, Kimberly Cartmill, Elana Sydney, Donald P. Kotler

**Affiliations:** 1 Department of Internal Medicine, Jacobi Medical Center, Albert Einstein College of Medicine, Bronx, NY USA; 2 Department of Internal Medicine, Metropolitan Hospital/New York Medical College, New York, NY USA; 3 Division of Gastroenterology and Hepatobiliary Diseases, Metropolitan Hospital/New York Medical College, New York, NY USA; 4 Gastroenterology, Hepatology, and Nutrition Service, Memorial Sloan Kettering Cancer Center, New York, NY USA; 5 Department of Internal Medicine, North Central Bronx Hospital, New York, NY USA; 6 Division of Gastroenterology, Jacobi Medical Center/Albert Einstein College of Medicine, 1400 Pelham Parkway South, Bronx, NY 10461 USA

**Correction: Journal of Gastrointestinal Cancer (2025) 56:69**

10.1007/s12029-025-01187-y

Recently Published the Article “Fecal Immunochemical Testing for Colorectal Cancer Prevention in Two Public Hospitals 10.1007/s12029-025-01187-y” on Journal of Gastrointestinal Cancer. We would like to reach out for 2 issues:

1. Correction in Discussion
  In this article, we cited a Danish study on adherence to colonoscopy at the end of Paragraph 4 of Discussion.
  “Our study revealed that despite these advantages, adherence remains a major obstacle in CRC screening with FIT. Previous studies in the Midwest of the US, California, China, and Denmark also reported a similar colonoscopy adherence rate of 40–50% after a positive FIT [33–36].”
  The citation 34 is the Danish study:
  “34. Jørgensen SF, Larsen PT, Erichsen R, Andersen B, Rebolj M, Njor S. Adherence to follow-up and resource use after abnormal FIT-screening: evaluation of the Danish colorectal cancer screening program. Endosc Int Open. 2024;12(5):E649-e658.”
  The correct statement should be
  “Previous studies in the Midwest of the US, California, and China also reported a similar colonoscopy adherence rate of 40–50% after a positive FIT.”
  The citation 34 should also be removed.
  We sincerely apologize for this mistake. Please kindly let us know if further actions should be taken from our side
2. Figure accuracy
  Figure 1 of the published article did not look like the submitted figure. The lines and boxes are absent and only the texts are present. We uploaded the correct files in both pdf and jpg format.

The original article has been corrected.

